# SOX2 is frequently downregulated in gastric cancers and inhibits cell growth through cell-cycle arrest and apoptosis

**DOI:** 10.1038/sj.bjc.6604193

**Published:** 2008-02-12

**Authors:** T Otsubo, Y Akiyama, K Yanagihara, Y Yuasa

**Affiliations:** 1 Department of Molecular Oncology, Graduate School of Medicine and Dentistry, Tokyo Medical and Dental University, 1-5-45, Yushima, Bunkyo-ku, Tokyo 113-8519, Japan; 2 Central Animal Laboratory, National Cancer Center Research Institute, 5-1-1 Tsukiji, Chuo-ku, Tokyo 104-0045, Japan

**Keywords:** SOX2, gastric cancer, cell cycle, apoptosis, DNA methylation

## Abstract

SOX transcription factors are essential for embryonic development and play critical roles in cell fate determination, differentiation and proliferation. We previously reported that the SOX2 protein is expressed in normal gastric mucosae but downregulated in some human gastric carcinomas. To clarify the roles of SOX2 in gastric carcinogenesis, we carried out functional characterisation of SOX2 in gastric epithelial cell lines. Exogenous expression of SOX2 suppressed cell proliferation in gastric epithelial cell lines. Flow cytometry analysis revealed that SOX2-overexpressing cells exhibited cell-cycle arrest and apoptosis. We found that SOX2-mediated cell-cycle arrest was associated with decreased levels of cyclin D1 and phosphorylated Rb, and an increased p27^Kip1^ level. These cells exhibited further characteristics of apoptosis, such as DNA laddering and caspase-3 activation. *SOX2* hypermethylation signals were observed in some cultured and primary gastric cancers with no or weak *SOX2* expression. Among the 52 patients with advanced gastric cancers, those with cancers showing *SOX2* methylation had a significantly shorter survival time than those without this methylation (*P*=0.0062). Hence, SOX2 plays important roles in growth inhibition through cell-cycle arrest and apoptosis in gastric epithelial cells, and the loss of SOX2 expression may be related to gastric carcinogenesis and poor prognosis.

Gastric cancer is the second most frequent cause of death from cancer in both sexes in the world (Shibuya *et al*, 2002). Although several environmental factors, such as *Helicobacter pylori* infection, excessive intake of salt and low intake of vegetables and fruit, have been linked with gastric carcinogenesis, the molecular mechanisms underlying gastric carcinogenesis are poorly understood yet (Peek and Blaser, 2002; Yuasa, 2003). In recent years, the relation between anomalous expression of transcription factors, such as *SOX*, *CDX* and *GATA*, and carcinogenesis has been focused on in various cancers, including gastric cancer (Akiyama *et al*, 2003; Yuasa, 2003).

The *SOX* gene family has been identified through their homology to the high-mobility group (HMG) box of sex-determining region Y, and encodes transcription factors that bind to DNA through a HMG domain and play critical roles in cell fate determination, differentiation and proliferation (Wegner, 1999; Kamachi *et al*, 2000; Wilson and Koopman, 2002). SOX2 belongs to group B of the SOX family, and is encoded by a single exon gene that is highly conserved in vertebrates. In fact, human SOX2 shares a 98% overall similarity with the mouse protein, and 95% with the chicken homologue cSox2 (Pevny and Lovell-Badge, 1997). *Sox2* mRNA has been detected in the brain, retina, lung and stomach in adult mouse tissues (Yuan *et al*, 1995). In chicken, cSox2 protein is expressed in epithelial cells of the proventriculus and gizzard (Ishii *et al*, 1998). The caudal limit of cSox2 expression coincides with that of the region competent for proventricular differentiation and to the rostral limit of expression of CdxA, a chicken member of the caudal-related homeobox gene family (Ishii *et al*, 1998). In the human digestive tract, SOX2 expression is found in the stomach, including fundic and pyloric mucosae, but not in the intestine, as observed in chicken (Tsukamoto *et al*, 2004). Furthermore, our previous study demonstrated that SOX2 protein levels were reduced in gastric carcinoma tissues compared with normal gastric epithelia (Li *et al*, 2004). Although decreased expression of SOX2 might be related with carcinogenesis of the human gastric epithelium, the roles of SOX2 in gastric carcinogenesis remain unclear.

Epigenetic gene silencing through DNA methylation is one of the important steps during carcinogenesis, including that in the stomach (Esteller *et al*, 2001; Jones and Baylin, 2002). To date, it has been reported that a large number of tumour-related genes were epigenetically silenced in human gastric cancers (Akiyama *et al*, 2003; Wen *et al*, 2006). Recently, it was reported that *SOX18*, a member of the SOX family, was methylated in lung carcinoma (Dammann *et al*, 2005). Other SOX factors, such as SOX4 and SOX9, have been reported as tumour-suppressive proteins in bladder (Aaboe *et al*, 2006), breast (Afonja *et al*, 2002), prostate (Drivdahl *et al*, 2004) and colon carcinomas (Jay *et al*, 2005).

However, it is not clear whether or not SOX2 has tumour-suppressive function in gastric cells. Here, to determine how SOX2 is involved in gastric carcinogenesis, we carried out functional characterisation of SOX2 in human and rat gastric epithelial cell lines. We further investigated the *SOX2* methylation status in human cultured and primary gastric cancer cells to clarify the mechanisms underlying the loss of SOX2 expression in gastric cancers.

## MATERIALS AND METHODS

### Cell lines and tissue samples

Ten human gastric cancer cell lines (MKN7, MKN45, MKN74, NUGC3, NUGC4, GCIY, TGBC11TKB, KATOIII, HSC58 and HSC59) were obtained as described previously (Tani *et al*, 2007). Rat gastric epithelial cell line OUMS37 was obtained from Dr Masayoshi Namba (Okayama University Medical School, Japan) (Pu *et al*, 1999). All the cell lines were cultured in appropriate medium. For demethylation studies, cells were daily treated with 5 *μ*M 5-aza-2′-deoxycytidine (Sigma, St Louis, MO, USA) for 48 h (Wen *et al*, 2006). A total of 74 primary gastric carcinoma tissue samples and corresponding noncancerous gastric mucosae were obtained as described previously (Wen *et al*, 2006). Informed consent was obtained from all subjects, and the study was approved by the institutional review committee. Genomic DNA was extracted using the standard phenol–chloroform procedure. Total RNA was extracted by using Trizol reagent (Invitrogen, Carlsbad, CA, USA) and treated with DNA-*free*™ (Ambion Inc., Austin, TX, USA).

### RT–PCR

For single-stranded cDNA synthesis, 1 *μ*g (for cell lines) or 2 *μ*g (for tissue samples) of total RNA was reverse transcribed using a Superscript II kit (Invitrogen). The amplification was performed by denaturation at 94°C for 4 min, followed by 21–38 cycles of 1 min each at 94°C, 55–64°C and 72°C, and a final extension at 72°C for 5 min. Primer sequences used for all genes are available upon request.

### Quantitative real-time RT–PCR

Quantitative real-time RT–PCR was carried out using LightCycler FastStart DNA Master SYBR Green I (Roche Diagnostic, Mannheim, Germany) and LyghtCycler software version 3.5 (Roche Diagnostic), according to the manufacturer's instructions. As an internal control, glyceraldehyde-3-phosphate dehydrogenase (*GAPDH*) was quantified using LightCycler Primer Set (Search LC GmbH, Heidelberg, Germany). Primer sequences used for *SOX2* are available upon request. The Second Derivative Maximum method was performed for the determination of concentration using LightCycler software version 3.5 (Roche Diagnostic).

### Adenovirus vector generation and infection

To generate the Ad-SOX2 vector, the human *SOX2* gene was subcloned into the pAdTrack-CMV shuttle vector (He *et al*, 1998) from the pME18S-SOX2 vector (Li *et al*, 2004). The Ad-GFP vector without any insert was generated as a control vector. The virus titre was determined by using Adeno-X™ Rapid Titer Kit (TAKARA BIO INC., Shiga, Japan), and infection was performed with the optimum MOI (ifu/cell) for each cell line to give at least 70% green fluorescent protein (GFP)-positive cells with minimal to no cytotoxicity.

### Small interfering RNA transfection

MKN45 and TGBC11TKB cells (5 × 10^5^ cells/well) were transfected with *SOX2* small interfering RNA (siRNA) (Sigma) to give a final concentration of 50 nM by using MicroPorator MP-100 (Digital Bio Technology, Seoul, Korea), according to the manufacturer's instructions. At 48 h after transfection, cells were harvested for western blot analysis. The nonspecific siRNA was used as a control (Neg control siRNA/Alexa Fluor 488, QIAGEN, Valencia, CA, USA).

### Western blot

Western blot analyses were performed as described previously (Li *et al*, 2004). The primary antibodies used were rabbit anti-SOX2 (1 : 1000; Chemicon International, Temecula, CA, USA; Li *et al*, 2004), mouse anti-cyclin D1 (1 : 200; Novocastra Laboratories Ltd, Newcastle, UK), rabbit anti-phospho (Ser780)-Rb (1 : 1000; Cell Signaling Technology, Danvers, MA, USA), mouse anti-p27^Kip1^ (1 : 2500; BD Transduction Laboratories, Franklin Lakes, NJ, USA), mouse anti-PARP (1 : 2000; Sigma), mouse anti-caspase-3 (1 : 1000; Cell Signaling Technology), rabbit anti-cleaved caspase-3 (1 : 1000; Cell Signaling Technology), mouse anti-p21^Cip1^ (1 : 100; Santa Cruz Biotechnology, Santa Cruz, CA, USA) and mouse anti-*α*-tubulin (1 : 200; Santa Cruz Biotechnology). The secondary antibodies used were alkaline phosphatase-conjugated anti-mouse IgG or anti-rabbit IgG (1 : 2000; Bio-Rad Laboratories, Hercules, CA, USA). Blots were developed with Immun-Star™ AP Substrate (Bio-Rad).

### Cell proliferation assay

OUMS37 cells were plated at 5 × 10^2^ cells/well, and NUGC3 and GCIY cells at 1 × 10^3^ cells/well on 96-well plates. After culturing for 24 h, cells were infected with Ad-SOX2 or control Ad-GFP at the optimal MOI. Cell proliferation was evaluated on days 1, 3 and 5 after infection by determining the number of cells with Cell Proliferation Reagent WST-1 (Roche Diagnostic), according to the manufacturer's instructions.

### Flow cytometry

At 24, 48 and 72 h after infection, OUMS37 cells were harvested and washed with PBS, followed by fixation with 70% ethanol overnight at −20°C. After washing with 3% BSA/PBS twice, the cells were resuspended in PBS containing 50 *μ*g ml^−1^ propidium iodide and 10 *μ*g ml^−1^ RNase A for 30 min at room temperature in the dark. Samples were analysed for DNA content by flow cytometry. The cell-cycle phases were analysed using CELLQuest software (Becton Dickinson, San Jose, CA, USA).

### APOPercentage Apoptosis assay

OUMS37 cells were plated at 2.5 × 10^3^ cells/well on 96-well plates. After culturing for 24 h, the cells were infected with Ad-SOX2 or control Ad-GFP at the optimal MOI. The culture medium was replaced with fresh medium containing APOPercentage Dye (Biocolor, Belfast, Northern Ireland) at 24, 48 and 72 h after infection, followed by incubation for 1 h at 37°C/5% CO_2_. After replacing the culture medium containing APOPercentage Dye with PBS, apoptotic cells exhibiting purple-red staining were counted under a phase contrast microscope in 10 random fields, and their percentage in total cells was calculated.

### DNA ladder assay

At 48 and 72 h after infection, OUMS37 cells were harvested and resuspended in lysis buffer (50 mM Tris-HCl (pH 8.0), 10 mM EDTA, 0.3% Triton X-100) for 30 min on ice. After centrifugation at 15 000 rpm for 5 min, the supernatant was treated with RNase (100 *μ*g ml^−1^) for 30 min at 55°C and then with proteinase K (400 *μ*g ml^−1^) for another 1 h at 55°C. The cell lysates were extracted with phenol–chloroform. DNA was precipitated with ethanol, and electrophoresed on 2% agarose gels.

### Methylation analysis

Bisulphite treatment of genomic DNA was carried out using Methylamp™ One-Step DNA Modificaion Kit (Epigentek Group Inc., Brooklyn, NY, USA), according to the manufacturer's instructions. Methylation-specific PCR (MSP) and bisulphite-sequencing analyses were performed as described previously (Wen *et al*, 2006). Primer sequences used for all analyses are available upon request.

### Statistical analysis

*χ*^2^-test was used to compare the values for the test and control samples, and a value of *P*<0.05 was taken as significant. Patient survival was calculated from the date of surgery until death or the date of last follow-up. Survival was evaluated by the Kaplan–Meier method, and the differences were evaluated with the log-rank test.

## RESULTS

### Frequent downregulation of *SOX2* mRNA in gastric cancer cell lines and primary gastric carcinoma tissues

**Figure 1:**
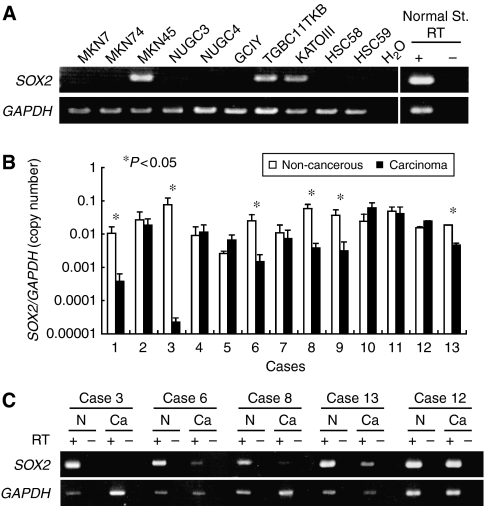
*SOX2* mRNA expression in gastric cancer cell lines and primary gastric carcinoma tissues. (**A**) RT–PCR analysis of *SOX2* mRNA levels in 10 gastric cancer cell lines and the normal stomach mucosae (normal st.). *GAPDH* expression was used as an internal loading control. RT (+ or −) indicates reverse transcriptase added or not, and H_2_O indicates no RNA added. (**B**) Quantitative real-time RT–PCR analysis of *SOX2* mRNA levels in primary gastric carcinoma samples and corresponding noncancerous gastric mucosae from the same patients. *SOX2* expression levels were normalised by internal *GAPDH* expression. The assay was performed in triplicate, and the bars indicate s.d. (**C**) Representative results of the endpoint RT–PCR of *SOX2* in primary gastric carcinomas (lanes Ca) and noncancerous gastric mucosae (lanes N).

To determine *SOX2* expression levels, we performed RT–PCR analysis in 10 human gastric cancer cell lines and the normal stomach mucosae. Among the 10 cell lines we investigated, 7 showed low or undetectable levels of *SOX2* mRNA compared with the normal stomach mucosae (Figure 1A). To assess *SOX2* expression levels in primary gastric cancer samples, we examined the expression levels of *SOX2* mRNA using quantitative real-time RT–PCR in primary gastric carcinoma tissues and corresponding noncancerous mucosae. Significant reductions of *SOX2* expressions were observed in 6 out of 13 cases (cases 1, 3, 6, 8, 9 and 13) (Figure 1B). Representative results of the endpoint RT–PCR are shown in Figure 1C.

### Exogenous SOX2 inhibits proliferation of gastric epithelial cell lines

**Figure 2:**
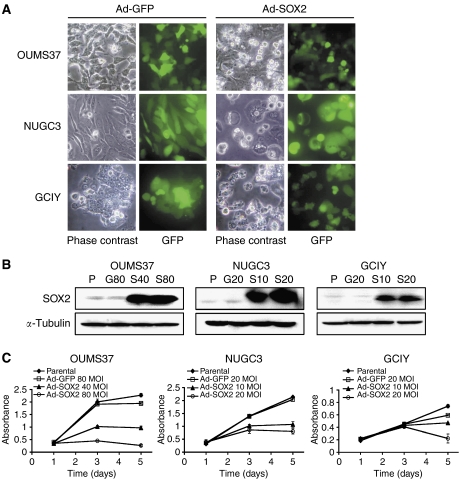
Effects of SOX2 overexpression on the proliferation of gastric epithelial cell lines. (**A**) Morphological changes in OUMS37, NUGC3 and GCIY cell lines after infection with the Ad-SOX2 or Ad-GFP. The MOI of the adenovirus vectors were 80 for OUMS37 and 20 for NUGC3 and GCIY, respectively. The left and right panels show the phase contrast micrographs appearance and fluorescent micrographs for GFP expression in the same fields. (**B**) Western blot analysis of SOX2 protein expression after adenovirus-mediated overexpression in OUMS37, NUGC3 and GCIY cell lines. SOX2 protein expression levels were compared among parental cells (P), Ad-GFP-infected cells (MOI: G80 for OUMS37, and G20 for NUGC3 and GCIY, respectively) and Ad-SOX2-infected cells (MOI: S40 and S80 for OUMS37, and S10 and S20 for NUGC3 and GCIY, respectively) at 48 h after adenovirus infection. *α*-tubulin expression was used as a protein loading control. (**C**) *In vitro* cell proliferation assay after SOX2 overexpression in gastric cell lines. The number of viable cells was measured by WST-1 assay of 1, 3 and 5 days after adenovirus infection. The assay was performed in triplicate, and bars indicate s.d.

To perform functional analysis of SOX2, we transiently expressed exogenous SOX2 in two human gastric cancer cell lines (NUGC3 and GCIY) and OUMS37 cells derived from rat gastric epithelia by using an adenovirus system. According to GFP expression, over 70% of the cells were infected with the vectors (Figure 2A). Although these cell lines showed basally low levels of SOX2 expression, abundant SOX2 protein was detected after Ad-SOX2 infection but not after the control Ad-GFP infection (Figure 2B). We found that SOX2-overexpressing cells exhibited dramatic morphological changes, that is, a round shape and floating, but such changes were not found in the control Ad-GFP-infected cells (Figure 2A).

To determine the proliferation rates after overexpression of SOX2 in gastric cell lines, growth curves were generated for each cell line by means of WST-1 assay over a 5-day period. We found that all SOX2-overexpressing cell lines showed significant growth inhibition compared with the control Ad-GFP-infected cells (Figure 2C).

### SOX2 induces cell-cycle arrest in gastric cell lines

**Figure 3:**
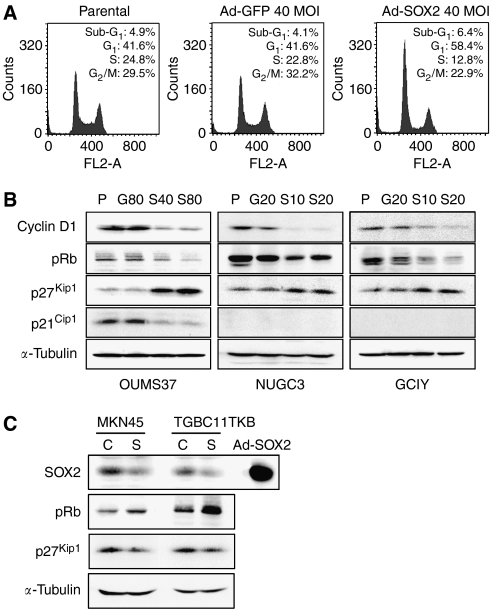
Effects of SOX2 on cell cycle. (**A**) Cell-cycle analysis of parental, Ad-GFP- and Ad-SOX2-infected OUMS37 cells at 24 h after infection. DNA content was measured by propidium iodide staining on flow cytometry. The percentages of cell-cycle phases are shown in each panel. (**B**) Differential expression of the cell-cycle-regulatory proteins associated with SOX2 overexpression. Western blot analysis was performed to compare expression levels of cyclin D1, pRb, p27^Kip1^ and p21^Cip1^ proteins among parental, Ad-GFP- and Ad-SOX2-infected cells. Expression of *α*-tubulin was used as a protein loading control. (**C**) Western blot analysis of cell-cycle-regulatory proteins expression after transfection with SOX2 siRNA (S) or control siRNA (C) in MKN45 and TGBC11TKB cell lines. Ad-SOX2-infected NUGC3 cells (Ad-SOX2) were used as a positive control of the SOX2 protein band. Expression of *α*-tubulin was used as a protein loading control.

To clarify the mechanisms underlying growth inhibition by SOX2 in gastric cell lines, we performed cell-cycle analysis by flow cytometry on cells stained with propidium iodide. We chose OUMS37 cells, for which the growth inhibition by SOX2 overexpression was the highest among the three cell lines (Figure 2C). At 24 h after infection, the Ad-SOX2-infected OUMS37 cells showed a higher proportion of cells in G_1_ phase (58.4%), compared with the control cells (41.6% for parental cells and Ad-GFP-infected cells), and concomitant decreases in the proportions of cells in S and G_2_/M phases (Figure 3A).

To investigate potential molecular mechanisms for SOX2-induced G_1_ arrest in gastric epithelial cells, we next explored the effects of SOX2 on expression of cell-cycle-regulatory proteins. Western blot analysis showed that the protein levels of cyclin D1 and phosphorylated Rb were decreased, whereas that of p27^Kip1^ was increased in the Ad-SOX2-infected OUMS37 cells, compared with control cells (Figure 3B). These changes in cell-cycle-regulatory proteins were also seen in human gastric cancer cell lines NUGC3 and GCIY on SOX2 overexpression (Figure 3B). Unexpectedly, the protein level of p21^Cip1^ was significantly decreased in the Ad-SOX2-infected OUMS37 cells compared with the control cells, whereas p21^Cip1^ proteins were undetectable levels in the NUGC3 and GCIY cell lines (Figure 3B).

We further performed the knockdown of endogenous SOX2 by siRNA in the SOX2-positive human gastric cancer cell lines. After transfection of SOX2 siRNA in MKN45 and TGBC11TKB cells, expression of endogenous SOX2 protein was reduced in both cell lines compared with control nonspecific siRNA-transfected cells (Figure 3C). Consequently, phosphorylated Rb level was significantly increased, whereas p27^Kip1^ was decreased in both cell lines compared with control cells (Figure 3C).

### SOX2 induces apoptosis in gastric cells through a caspase-3-dependent pathway

**Figure 4:**
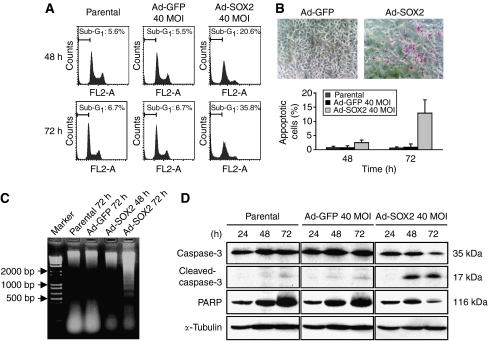
Analyses of apoptosis associated with SOX2 overexpression in OUMS37 cells. (**A**) Percentage of the sub-G_1_ DNA fraction in OUMS37 cells at 48 and 72 h after infection. DNA was stained with propidium iodide and the sub-G_1_ fraction was measured by flow cytometry. The percentages of the sub-G_1_ fractions, indicated by the bars, are shown in each panel. (**B**) Apoptosis detection by APOPercentage Apoptosis assay. Top panels: phase contrast micrographs indicate representative areas of the control (Ad-GFP 40MOI) and SOX2-overexpressing (Ad-SOX2 40MOI) OUMS37 cells at 72 h after adenovirus infection. Bottom panel: purple-red-stained apoptotic cells were counted in multiple randomly selected fields and the percentage (per total cells) was determined (columns, mean of 10 fields; bars, s.d.). (**C**) DNA ladder assay by agarose gel electrophoresis. DNA extracted from the control (parental and Ad-GFP 40MOI for 72 h) and SOX2-overexpressing (Ad-SOX2 40MOI for 48 and 72 h) OUMS37 cells was electrophoresed on a 2% agarose gel and detected by ethidium bromide staining. Arrows indicate the molecular weights of DNA base pairs. (**D**) Detection of activated caspase-3 in SOX2-overexpressing OUMS37 cells on western blot analysis. The reduction of caspase-3 and accompanying appearance of cleaved-caspase-3 indicate activation of caspase-3. The reduction of a caspase-3 substrate, PARP, also reflects activation of caspase-3. Expression of *α*-tubulin was used as a protein loading control.

As shown in Figure 2A, dramatic morphological changes that seemed to be hallmarks associated with apoptosis were observed in SOX2-overexpressing gastric cell lines. To determine whether apoptosis occurred in SOX2-overexpressing cells, we performed flow cytometry analysis. As shown in Figure 4A, a significant increase in the percentage of cells with a hypodiploid (sub-G_1_) DNA content was observed in SOX2-overexpressing OUMS37 cells (20.6 and 35.8% for 48 and 72 h, respectively) in comparison with the control Ad-GFP-infected cells (5.5 and 6.7% for 48 and 72 h, respectively).

To further characterise the apoptosis, we performed APOPercentage Apoptosis assay in the Ad-SOX2-infected OUMS37 cells. The APOPercentage Apoptosis assay is a detection and measurement system for monitoring the occurrence of apoptosis, involving APOPercentage Dye that is selectively imported by cells undergoing apoptosis with modified cell membrane phospholipid composition, showing purple-red staining due to the accumulation of the APOPercentage Dye. As shown in upper panels of Figure 4B, there were many purple-red-stained apoptotic cells in the Ad-SOX2-infected OUMS37 cells, but not in the control Ad-GFP-infected cells. The percentage of apoptotic cells per total cells was determined, and a significant increase in apoptotic cells was observed in Ad-SOX2-infected OUMS37 cells (2.5 and 12.9% for 48 and 72 h, respectively) compared with the control Ad-GFP-infected cells (0.7 and 0.9% for 48 and 72 h, respectively; Figure 4B, bottom). We further performed the DNA ladder assay, and observed a typical DNA ladder formation in the Ad-SOX2-infected OUMS37 cells, but not in the control Ad-GFP-infected and parental OUMS37 cells (Figure 4C).

To confirm whether these cell deaths were caspase dependent or independent, we examined the effects of SOX2 on the activation of caspase-3, an effector caspase. Western blot analysis showed that the protein level of the inactivated form of caspase-3 (35 kDa) was decreased, while the activated form of caspase-3 (17 kDa) appeared, and the caspase substrate PARP was diminished in the Ad-SOX2-infected OUMS37 cells at 48 and 72 h after infection compared with the control Ad-GFP-infected and parental cells (Figure 4D).

### Epigenetic silencing of the *SOX2* gene in gastric cancer cell lines

**Figure 5:**
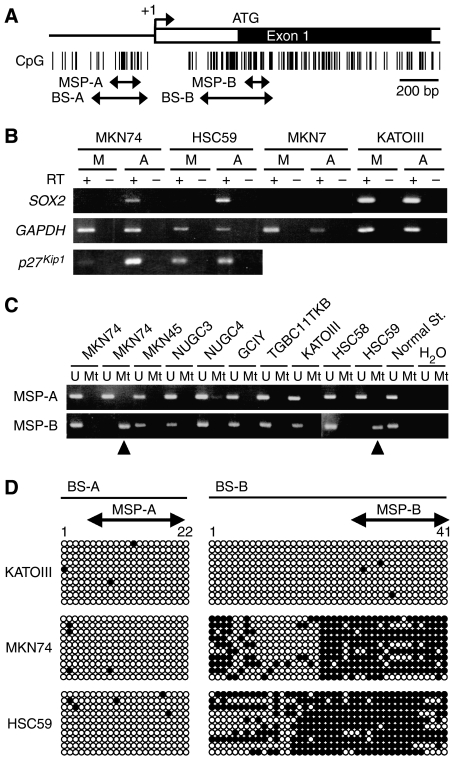
Methylation status of *SOX2* in gastric cancer cell lines. (**A**) Schematic representation of the human *SOX2* gene. Open and closed boxes indicate the untranslating and coding regions, respectively, and an arrow denotes the transcription start site (+1). Vertical bars show CpG sites. Arrows below the CpG sites indicate the regions subjected to MSP (MSP-A and MSP-B) and bisulphite sequencing (BS-A and BS-B). (**B**) Representative results of the demethylation analysis of *SOX2*. Gastric cancer cell lines were treated with (lanes A) or without (lanes M; mock) 5-aza-2′-deoxycytidine (5 *μ*M), and *SOX2* expression was examined by RT–PCR as described in Figure 1A. *p27*^*Kip1*^ expression was also examined. (**C**) MSP analyses in gastric cancer cell lines and the normal stomach. The primer region is depicted in panel A. Bands in the ‘Mt’ lanes, as shown by arrowheads, are PCR products obtained with methylation-specific primers; those in the ‘U’ lanes are obtained with unmethylated-specific primers. (**D**) Sodium bisulphite DNA sequencing of *SOX2* in gastric cancer cell lines (KATOIII, MKN74 and HSC59). The analysed regions are shown in panel A. Each horizontal row of circles represents analysis, in single clone of bisulphite-treated DNA, of 22 or 41 CpG sites (for BS-A or BS-B, respectively) contained in the regions. Solid and open circles represent methylated and unmethylated CpG sites, respectively.

Next, we tried to clarify the mechanisms underlying the loss of *SOX2* expression in gastric cancers. As there is a dense CpG island in the 5′ and exon regions of *SOX2* (Figure 5A), we analysed DNA methylation status of *SOX2* in human gastric cancer cell lines. To initially study the epigenetic status of *SOX2* in gastric cancer cell lines, we used a demethylating agent, 5-aza-2′-deoxycytidine. RT–PCR analysis revealed that the basally silent *SOX2* gene was re-expressed in MKN74 and HSC59 cell lines after 5-aza-2′-deoxycytidine treatment, whereas all the other cell lines, such as MKN7, did not restore the expression of *SOX2* with this drug treatment (Figure 5B). Furthermore, in MKN74 and HSC59 cell lines, restoration of *SOX2* by 5-aza-2′-deoxycytidine reinduced expression of *p27*^Kip1^ that was upregulated in the Ad-SOX2 infected cells, although the level of induction was weak in HSC59 cells (Figure 5B). On the other hand, the treatment with a histone deacetylase inhibitor, trichostatin A (TSA), could not restore *SOX2* expression in these cell lines except MKN7 with a weak restoration (data not shown).

As shown in Figure 5A, we designed two MSP primer sets (MSP-A and MSP-B). Among the 10 gastric cancer cell lines, MKN74 and HSC59 exhibited the hypermethylation signals only with the MSP-B primer set (Figure 5C). In contrast, normal stomach mucosae exhibited an unmethylated status using both primer sets (Figure 5C). Bisulphite sequencing data of MKN74, HSC59 and KATO-III are consistent with the ones of MSP analyses (Figure 5D), indicating that the methylation status using the MSP-B primers is strongly related to *SOX2* expression patterns.

### *SOX2* methylation status in primary gastric carcinoma tissues

**Figure 6:**
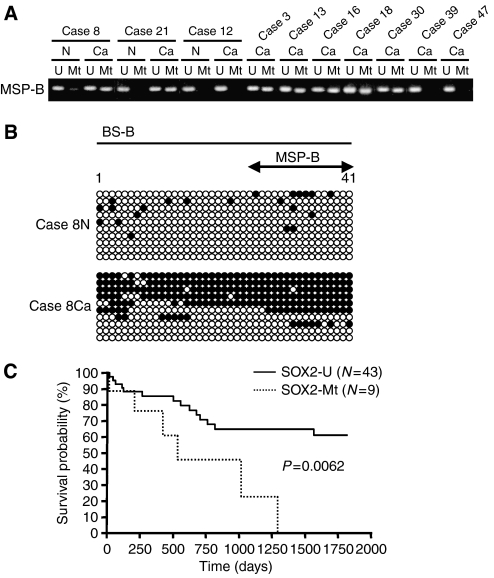
Methylation status of *SOX2* in primary gastric carcinoma tissues, and its involvement in prognosis. (**A**) Representative results of MSP analysis of *SOX2* in primary gastric carcinomas (lanes Ca) and corresponding noncancerous gastric mucosae (lanes N). All analyses were carried out by using MSP-B primers (shown in Figure 5A). (**B**) Sodium bisulphite DNA sequencing of *SOX2* in noncancerous gastric mucosae (Case 8N) and primary gastric carcinoma (Case 8Ca) tissue samples. The analysed regions (BS-B) are shown in Figure 5A. Each horizontal row of circles represents analysis, in single clone of bisulphite-treated DNA, of 41 CpG sites contained in the regions. Solid and open circles represent methylated and unmethylated CpG sites, respectively. (**C**) Kaplan–Meier survival analysis of *SOX2* methylation in 52 advanced gastric cancer patients. Patients with *SOX2* methylation (Mt; dotted) had a significantly poorer outcome than those without methylation (U; solid) (*P*=0.0062).

To investigate the relationship between *SOX2* expression levels and methylation status in primary gastric carcinoma tissues, we initially examined *SOX2* methylation status in 13 primary gastric cancer tissues and corresponding noncancerous mucosae (cases 1–13) prepared from frozen samples, whose expression levels of *SOX2* were already examined (Figure 1B). Among the 13 cases, the hypermethylation signals of *SOX2* were observed in 3 cases (cases 3, 8 and 13), in which *SOX2* expression was downregulated, whereas all the other cases, such as case 12, revealed the unmethylated status by the MSP analysis (Figure 6A). Although a noncancerous sample (Figure 6A, case 8N) displayed a weak methylation signal at 35 cycles of PCR condition, the methylation band disappeared in PCR cycles at 32 (data not shown) and bisulphite sequencing did not show any clone with dense methylation signals (Figure 6B, case 8N). Thus, we regarded this weak band in case 8N as negative, and all the 13 noncancerous tissues expressing *SOX2* were found to be methylation negative. On the other hand, densely methylated clones were observed in the *SOX2* downregulated primary gastric carcinoma tissue (Figure 6B, case 8Ca). These data strongly suggest the correlation between the *SOX2* hypermethylation and its reduced expression in primary gastric carcinoma tissues.

We subsequently investigated the methylation status of the additional cases (61 and 11 for gastric carcinomas and corresponding noncancerous gastric mucosae, respectively) from paraffin-embedded samples by the MSP analysis. The representative results of SOX2-methylation-positive (cases 16, 18, 21 and 30) and -negative (cases 39 and 47) cases are shown in Figure 6A. Altogether, 12 out of 74 (16.2%) primary gastric carcinoma tissues exhibited the hypermethylation signals of *SOX2*, whereas none of the 24 corresponding noncancerous tissues did (Fisher's exact probability test, *P*=0.027).

When we analysed a relationship between the *SOX2* methylation status and clinicopathological features of the gastric cancer cases, there was no significant correlation between the *SOX2* methylation status and sex, age, depth of tumour invasion and histological type (data not shown). However, among the 52 patients with advanced gastric cancers, Kaplan–Meier analysis demonstrated that those with cancers showing *SOX2* methylation had a significantly shorter survival than those without this methylation (*P*=0.0062; Figure 6C).

## DISCUSSION

We have previously reported that the SOX2 protein level was often reduced in human gastric carcinoma tissues on immunohistochemical analysis (Li *et al*, 2004). In the present study, we found that expression levels of *SOX2* mRNA were frequently downregulated in human gastric cancer cell lines and primary gastric carcinomas. These results are consistent with our previous studies, and indicate that downregulation of the SOX2 protein may occur at a transcriptional level in gastric cancer cells.

In the present study, we performed functional characterisation of SOX2 in gastric epithelial cell lines by adenoviral overexpression of SOX2, and we found that exogenous SOX2 inhibited cell proliferation through cell-cycle (G_1_) arrest and apoptosis. Generally, overgrowth due to the deviation from canonical cell cycle and/or loss of the propensity to undergo apoptosis are critical events in the development of cancer (Lowe and Lin, 2000; Sherr, 2000). Hence, our present data suggest that SOX2 plays a crucial role in gastric carcinogenesis as a tumour suppressor, and loss of SOX2 expression may cause gastric epithelial cells develop into carcinomas.

We demonstrated that decreased protein levels of cyclin D1 and phosphorylated Rb, and increased protein levels of p27^Kip1^ were associated with SOX2 overexpression. Moreover, knockdown of SOX2 by siRNA upregulated phosphorylated Rb and downregulated p27^Kip1^ in the two gastric cancer cell lines. These results are consistent with the increased proportion of G_1_ phase shown in the Ad-SOX2-infected OUMS37 cells. Downregulation of the *cyclin D1* mRNA and upregulation of the *p27*^Kip1^ mRNA were also observed after SOX2 overexpression (data not shown), indicating that these cell-cycle-regulating factors may be controlled by SOX2 at the transcriptional levels. It has been reported that *β*-catenin activates the transcription of *cyclin D1* through TCF-binding sites within the promoter, resulting in a direct effect on cell proliferation in colon carcinoma cells (Tetsu and McCormick, 1999), whereas SOX2 associates with *β*-catenin in osteoblasts and inhibits the activity of a Wnt-responsive reporter plasmid in HEK293 cells (Mansukhani *et al*., 2005). It is, thus, possible that SOX2 may function in the cell cycle through interaction with Wnt signals in gastric epithelial cells. On the other hand, our present data strongly suggest the association between SOX2 and p27^Kip1^ by means of overexpression and knockdown experiments. Recently, it has been reported that knockdown of SOX9, another SOX family member, induced cell-cycle arrest at G_1_ and upregulated p27^Kip1^ in prostate cancer cells (Wang *et al*., 2007), indicating that SOX9 positively regulates cell-cycle progression though inhibiting p27^Kip1^. However, it is controversial because other groups reported SOX9 as a suppressor of cell-cycle progression (Panda *et al*., 2001; Afonja *et al*, 2002; Drivdahl *et al*, 2004). Thus, further studies are necessary to clarify the role of SOX2 in the regulation of cell cycle.

We further elucidated that exogenous expression of SOX2 induced typical apoptosis, which accompanied by the caspase-3 activation, in gastric epithelial cells. Among the wide spectrum of apoptosis-regulatory genes, such as *Bax*, *Bcl-2*, *Puma*, *FasL*, and so on, no remarkable changes in expression of any genes were observed in Ad-SOX2-infected OUMS37 cells (data not shown). It has been reported that other SOX family members, such as SOX4 and SOX9, induce apoptosis in bladder, prostate and colon carcinomas (Drivdahl *et al*, 2004; Jay *et al*, 2005; Aaboe *et al*, 2006), being consistent with our finding. However, transcriptional targets of SOX family members involving apoptosis have been poorly understood yet. In the present study, p21^Cip1^ was found to be downregulated in SOX2-overexpressing OUMS37 cells, whose expression change has also been detected in mRNA levels (data not shown). This observation is surprising, because p21^Cip1^ is an inhibitor of cell-cycle progression and is known as a tumour suppressor (Harper *et al*, 1993). However, recent finding has shown that p21^Cip1^ is implicated in the antiapoptotic response to chemotherapy (Gartel and Tyner, 2002). As a negative regulator of apoptosis, p21^Cip1^ interacts with several caspases, such as caspase-3, caspase-8 and caspase-10 (Gartel and Tyner, 2002). Moreover, it was reported that upregulation of p21^Cip1^ confers resistance to apoptosis in human gastric cancer cell lines MKN28 and MKN45 (Liu *et al*, 2004). Meanwhile, it has been reported that p27^Kip1^ positively regulates, at least in part, the apoptotic response of gastric epithelial cells to *H. pylori* (Eguchi *et al*, 2004). As these reports are consistent with our present data, SOX2 may induce apoptosis through p21^Cip1^ and/or p27^Kip1^ in gastric epithelial cells.

In this study, we found that the exogenous SOX2 overexpression induced cell-cycle arrest at an early time point and apoptosis at a later time point in gastric epithelial cells. In the normal stomach, terminally differentiated epithelial cells stop proliferation and eventually culminate in apoptosis (Johnson, 1988). Our present results may therefore be similar to the normal cell differentiation process in the stomach. Furthermore, SOX2 has been found to upregulate the expression levels of gastric differentiation markers, *MUC5AC* and *pepsinogen A* (Li *et al*, 2004; Tani *et al*, 2007). These data, combined with the findings for antiproliferation effects of SOX2 in gastric epithelial cell lines, suggest that SOX2 may control the gastric epithelial cells differentiating into mature cells and its disruption may cause continual dividing, eventually leading to gastric cancer. However, further investigations, for example, generation of an animal model, are necessary to understand the precise SOX2 functions in the normal stomach differentiation.

Finally, we investigated the methylation status of *SOX2* in human gastric cancer cell lines and primary gastric carcinoma tissues. Two *SOX2* expression-negative gastric cancer cell lines (MKN74 and HSC59) exhibited the hypermethylation signals by MSP and bisulphite sequencing, and restored the *SOX2* expression after the demethylating agent treatment. Furthermore, the *SOX2* hypermethylation was more frequently observed in primary gastric carcinomas than corresponding noncancerous mucosae (16.2 *vs* 0%, respectively). Particularly in *SOX2*-downregulated cases, half of the cases (three out of six) showed the hypermethylation signals. These data strongly suggest that aberrant DNA methylation is one of the key mechanisms underlying *SOX2* downregulation in gastric cancers. Nevertheless, it is important to search for other mechanisms, such as histone modification, in gastric cancers, because several *SOX2* expression-negative cases did not show any DNA methylation. In fact, a gastric cancer cell line MKN7 weakly restored its expression after treatment with a histone deacetylase inhibitor TSA.

Importantly, we demonstrated that patients with cancers showing *SOX2* methylation had a significantly shorter survival time than those without its methylation. It has been reported that low expression levels of p27^Kip1^ protein are related with a poor outcome in many human gastric cancers (Mori *et al*, 1997). In addition, the expression of the cyclin D1 and p27^Kip1^ proteins is inversely correlated and is associated with a poor clinical outcome in human gastric cancer (Han *et al*, 1999). These findings, combined with our present data, suggest that downregulation of SOX2 through aberrant DNA methylation and resultant alteration of cyclin D1 and p27^Kip1^ protein levels may be associated with poor prognoses in gastric cancers, and that *SOX2* methylation can be a clinically useful prognostic marker for advanced gastric cancer patients.

In conclusion, we revealed that *SOX2* expression was frequently downregulated in gastric cancers, some of which were due to aberrant DNA methylation. Moreover, we also showed that exogenous SOX2 expression inhibited cell growth through cell-cycle arrest and apoptosis in gastric cells. We have demonstrated, for the first time, SOX2 as a tumour-suppressive gene in gastric cancers, and our findings may lead to new therapeutic approaches for gastric cancer.
